# Microglia and astrocytes attenuate the replication of the oncolytic vaccinia virus LIVP 1.1.1 in murine GL261 gliomas by acting as vaccinia virus traps

**DOI:** 10.1186/s12967-015-0586-x

**Published:** 2015-07-07

**Authors:** Christina Kober, Susanne Rohn, Stephanie Weibel, Ulrike Geissinger, Nanhai G Chen, Aladar A Szalay

**Affiliations:** Department of Biochemistry, Biocenter, University of Wuerzburg, Am Hubland, 97074 Würzburg, Germany; Rudolf Virchow Center for Experimental Biomedicine and Institute for Molecular Infection Biology, University of Wuerzburg, 97080 Würzburg, Germany; Department of Radiation Medicine and Applied Sciences, Rebecca and John Moores Comprehensive Cancer Center, University of California, San Diego, CA 92093 USA; Genelux Corporation, San Diego Science Center, 3030 Bunker Hill Street, San Diego, CA 92109 USA; Department of Anesthesia and Critical Care, University Hospital of Wuerzburg, Oberduerrbacher Str. 6, 97080 Würzburg, Germany

**Keywords:** GBM, VACV, Polarization, Microglia, Tumor microenvironment, BV-2, IMA2.1, OSC

## Abstract

**Background:**

Oncolytic virotherapy is a novel approach for the treatment of glioblastoma multiforme (GBM) which is still a fatal disease. Pathologic features of GBM are characterized by the infiltration with microglia/macrophages and a strong interaction between immune- and glioma cells. The aim of this study was to determine the role of microglia and astrocytes for oncolytic vaccinia virus (VACV) therapy of GBM.

**Methods:**

VACV LIVP 1.1.1 replication in C57BL/6 and Foxn1^nu/nu^ mice with and without GL261 gliomas was analyzed. Furthermore, immunohistochemical analysis of microglia and astrocytes was investigated in non-, mock-, and LIVP 1.1.1-infected orthotopic GL261 gliomas in C57BL/6 mice. In cell culture studies virus replication and virus-mediated cell death of GL261 glioma cells was examined, as well as in BV-2 microglia and IMA2.1 astrocytes with M1 or M2 phenotypes. Co-culture experiments between BV-2 and GL261 cells and apoptosis/necrosis studies were performed. Organotypic slice cultures with implanted GL261 tumor spheres were used as additional cell culture system.

**Results:**

We discovered that orthotopic GL261 gliomas upon intracranial virus delivery did not support replication of LIVP 1.1.1, similar to VACV-infected brains without gliomas. In addition, recruitment of Iba1^+^ microglia and GFAP^+^ astrocytes to orthotopically implanted GL261 glioma sites occurred already without virus injection. GL261 cells in culture showed high virus replication, while replication in BV-2 and IMA2.1 cells was barely detectable. The reduced viral replication in BV-2 cells might be due to rapid VACV-induced apoptotic cell death. In BV-2 and IMA 2.1 cells with M1 phenotype a further reduction of virus progeny and virus-mediated cell death was detected. Application of BV-2 microglial cells with M1 phenotype onto organotypic slice cultures with implanted GL261 gliomas resulted in reduced infection of BV-2 cells, whereas GL261 cells were well infected.

**Conclusion:**

Our results indicate that microglia and astrocytes, dependent on their activation state, may preferentially clear viral particles by immediate uptake after delivery. By acting as VACV traps they further reduce efficient virus infection of the tumor cells. These findings demonstrate that glia cells need to be taken into account for successful GBM therapy development.

## Background

Glioblastoma multiforme (GBM) is one of the most malignant form of brain cancer and the most frequent type of gliomas in adults [1]. Difficulties associated with the treatment of GBM are mainly due to the infiltrative nature of these tumors into the brain tissues as well as the function and morphology of the blood–brain barrier preventing most therapeutics to reach the tumor sites [1, 2]. The success rate of conventional therapy is low and the prognosis for GBM is very poor, with a median survival time of less than 15 months [3], therefore, more emphasis on the research and development regarding alternative therapeutical approaches is essential. In this respect, the use of oncolytic viruses is an innovative and encouraging strategy in cancer therapy [4–6]. Especially, vaccinia virus (VACV) is a very promising candidate in the field of oncolytic virotherapy of tumors [7–10]. Although oncolytic viruses can kill tumor cells grown in vitro with high efficiency, they often exhibit reduced replication capacity in vivo, which results in a low therapeutic efficiency in clinical trials [11]. These findings suggest that physiological aspects of the tumor microenvironment and host defense mechanisms decrease the virus’ therapeutic potential. Continued preclinical research aimed to understand and overcome virus inhibitory physiological aspects is necessary. Two cell types present in brain tissues are suspected to be involved in the clearance of viral particles in the early stages of antitumor virotherapy. On the one hand microglial cells, the resident macrophages of the brain, quickly respond to a whole range of pathologic stimuli, e.g. virus infection [12–14]. At the same time, they exert an important neuroprotective role, for example, via phagocytosis of cellular debris after tissue injury, and they act as antigen-presenting cells in adaptive immune responses [15]. Like peripheral macrophages, microglia can exhibit either an acute inflammatory phenotype, referred to as classically activated or M1 microglia or an anti-inflammatory phenotype, also called alternatively activated or M2 microglia [16, 17]. Malignant gliomas may consist of up to 30% microglia [12, 18–20]. Interestingly, the local environment seems to determine the phenotype exhibited by microglia/macrophages. Gliomas are found to actively suppress antitumor activity of microglia e.g. by secretion of a variety of cytokines [21], which does result in promotion of the M2 phenotype and suppression of the M1 phenotype [22]. Several reports have shown a correlation between presence of microglial cells with M2 phenotype in gliomas and increasing tumor growth and poor prognosis [21, 23, 24]. In contrast, cells with the M1 phenotype are expected to be predominantly present at injection sites and also during the initial phases of tumor growth [24–26]. However, so far no detailed characterization of cells with M1 and M2 phenotypes has been conducted during glioma development [21]. In addition to microglia, astrocytes are also recruited to the tumor sites playing a role in the repair and scarring process following traumatic injuries [27, 28]. Astrocytes are derived from a heterogeneous population of progenitor cells present in the neuroepithelium of the developing CNS [27]. They are a heterogeneous population of cells thought to be involved in almost all aspects of brain functions, e.g. in biochemical support of endothelial cells that form the blood–brain barrier, in providing of nutrients to nervous tissues, as well as in maintenance of extracellular ion balance and transmitters [29, 30].

So far, there is little knowledge about the susceptibility of glial cells to VACV infection in adult brains and its possible outcome in the context of malignant gliomas. In the present study we examined whether murine glial cells are susceptible to VACV infection in cell culture and whether VACV can be used as a safe and efficient therapeutic agent in syngeneic mouse models for GBM. We further analyzed whether variable polarization of microglia and astrocytes have any impact on VACV infection. For studying oncolytic virotherapy in an aggressive tumor model such as murine GL261 glioma, we used the VACV LIVP 1.1.1, which is a less-attenuated strain in comparison to e.g. the extensively studied triple mutant oncolytic VACV GLV-1h68 [31–35]. LIVP 1.1.1 is a replication-competent wild-type isolate of the LIVP strain that has not been genetically modified but has a naturally occurring disruption at the thymidine kinase (*TK*) gene locus (Chen et al. manuscript in preparation) resulting in enhanced tumor specificity and reduced virulence [34, 36].

## Methods

### Cell culture

African green monkey kidney fibroblasts (CV-1) and the human primary glioblastoma cell line U87-MG were obtained from the American Type Culture Collection (ATCC). IMA2.1 cells were obtained from S. Schildknecht (University of Konstanz, Germany). These cells are immortalized murine cortical astrocytes that respond to certain stimuli similarly to primary astrocytes with upregulation of specific mRNA resulting in expression of proteins [37]. BV-2 cells were kindly provided by M. Karlstetter (University Hospital Cologne, Germany). This cell line was derived from raf/myc-immortalized murine neonatal microglia and is the most frequently used substitute for primary microglia [38–40]. The murine glioblastoma cell line GL261 was kindly provided by A. Pagenstecher (Department of Neuropathology, University Hospital of Marburg, Germany). The identity of GL261 cells was confirmed by DNA sequencing by the Leibniz Institute DSMZ—German Collection of Microorganisms and Cell Cultures (Braunschweig, Germany).

All cell lines were cultured in DMEM supplemented with antibiotic-solutions (100 U/ml penicillin G, 100 U/ml streptomycin, PAA Laboratories, Pasching, Austria) and with 10% fetal bovine serum (FBS; PAA Laboratories). Cultures were maintained and incubated at 37°C with 95% humidity and 5% CO_2_. Growth medium was changed every third day until confluence.

### Generation of U87-MG-RFP and GL261-RFP cells

The cDNA sequence of the red fluorescent protein (*mRFP1*) was inserted into the U87-MG and GL261 cell genome as described previously in detail for the cell line C33A [41]. After transfection of U87-MG or GL261 cells with mRFP-encoding lentiviruses followed by blasticidin selection (10 mg/ml, PAA Laboratories) one RFP-expressing clone was selected and stable RFP expression was confirmed in 98% of all cells by flow cytometry.

### Virus strains

LIVP 1.1.1 is a plaque purified isolate from the wild-type stock of LIVP strain (Lister strain, Institute of Viral Preparations, Moscow, Russia). The sequence analysis of LIVP 1.1.1 revealed the presence of different mutations in several viral genes including that of thymidine kinase (TK) [31, 32]. GLV-2b372 is a virus construct derived from the parental LIVP 1.1.1 virus strain, which carries the TurboFP635 expression cassette under the control of the vaccinia synthetic early/late promoter in the *TK* locus.

### Viral replication

Cells were grown in 24-well plates and infected with LIVP 1.1.1 at a multiplicity of infection (MOI) of 0.1. After 1 h of incubation at 37°C, the infection medium (infmed) was removed and replaced by fresh growth medium. After 2, 24, 48, 72, and 96 h cell pellets and supernatants were harvested. Following three freeze–thaw cycles, serial dilutions of the lysates were titrated by standard plaque assay on CV-1 cells. All samples were measured in duplicate.

For analysis of viral titers from tissues, brains were excised 1, 3 and 7 days after intracranial/intratumoral LIVP 1.1.1 injection, they were minced, and 1 ml of ice-cold phosphate buffered saline (PBS) was added. Samples were homogenized using a FastPrep homogenizer (Thermo Scientific, Karlsruhe, Germany).

### Cell viability assay

After 24 h in culture, cells were infected with LIVP 1.1.1 (MOI 1.0) for 1 h at 37°C. Afterwards the infection medium was replaced by fresh growth medium with or without cytokine supplement. The amount of viable cells after infection was determined by uptake of 3-(4,5-dimethylthiazol-2-yl)-2,5-diphenyltetrazolium bromide (MTT, Sigma-Aldrich, Taufkirchen, Germany). 24, 48, 72, or 96 h after virus infection the medium was replaced by 0.5 ml MTT solution at a concentration of 2.5 mg/ml MTT dissolved in DMEM without phenol red and incubated for 2 h at 37°C in the presence of 5% CO_2_. After removal of the MTT solution, 400 µl 1 N HCl diluted in isopropyl alcohol (Sigma-Aldrich) were added. The optical density was then measured at a wavelength of 570 nm. Uninfected cells were used as controls and were considered as 100% viable or were used to determine the cell density.

### Polarization experiments

For polarization experiments both 5 × 10^4^ BV-2 and IMA2.1 cells were plated in DMEM + 2% FBS in wells of 24-well plates and allowed to adhere for 20–24 h. 24 h prior to infection, cells were stimulated either with 1 µg/ml lipopolysaccharide (LPS, 026:B6 from E.coli, Sigma-Aldrich), LPS and rm-interferon-gamma (IFN-γ; 10 ng/ml, Immunotools GmbH, Oldenburg, Germany), rm-IFN-γ alone, rm-interleukin-4 (IL-4; 10 ng/ml, Immunotools GmbH), or basic fibroblast growth factor (bFGF, 100 ng/ml, Millipore, Schwalbach, Germany) in DMEM + 2% FBS. Cells were infected with LIVP 1.1.1 (MOI 1) for 1 h at 37°C. Infection medium was then replaced with fresh culture medium or culture medium supplemented with cytokines.

### Griess assay

Nitrite (surrogate marker for nitric oxide [NO]) was measured by using the Griess reagent system (Promega, Mannheim, Germany) according to the manufacturer’s instructions.

### Flow cytometry

For polarization experiments, BV-2 and IMA2.1 cells were incubated with rm-IL-4 or rm-IFN-γ as described above. Subsequently cells were trypsinized with 300 μl trypsin/EDTA (PAA Laboratories) until all cells were detached. The reaction was stopped by adding 600 μl of culture medium. Samples were centrifuged at 2,000 rpm for 3 min, 4°C and stained with the labeled monoclonal antibody anti-mouse MHCII-PE (Clone M5, eBioscience, Frankfurt, Germany) for 1 h at 4°C. Cells were washed once, resuspended in 200 µl PBS + 2% FBS, and analysis was done using the Accuri C6 Cytometer with FACS analysis software CFlow Version 1.0.227.4 (Accuri Cytometers, Inc., Ann Arbor, MI, USA).

### Apoptosis studies

Cells that have lost part of their DNA due to DNA fragmentation in late stage apoptosis are represented in sub-G1 peaks on DNA histograms [42]. For cell cycle analysis and detection of DNA fragmentation (sub-G1 phase) GL261 and BV-2 cells were cultured in 24‐well plates for 24 h and infected with LIVP 1.1.1 (MOI 0.5), respectively. Cell pellets were resuspended in a solution of 100 µl PBS + 2% FBS and 5 µl propidium iodide (PI; 1.0 µg/ml; Sigma-Aldrich). Prior to measurement, the suspension was frozen in liquid nitrogen and thawed at 37°C.

To enable distinguishing apoptotic cells from necrotic cells a PI exclusion assay was performed. Based on the status of membrane damage the uptake kinetics of PI differ and cells can be distinguished into healthy (PI-negative), apoptotic (PI-dim), and necrotic (PI-bright) cells [43, 44]. For PI staining the cells were incubated with 1 µl PI for 5 min at room temperature and were subsequently analyzed as described above.

### Western blot analysis

Cells were harvested and lysed in lysis buffer [50 mM Tris–HCl pH 7.4 (Carl Roth GmbH, Karlsruhe, Germany), 150 mM NaCl (Sigma-Aldrich), 1 mM EDTA (Sigma-Aldrich), 1% Triton X-100 (Applichem, Darmstadt, Germany), 1% sodium desoxycholate (Carl Roth GmbH), 0.1% SDS (Carl Roth GmbH), 1 tablet Complete (Roche Diagnostics, Mannheim, Germany)]. The cell lysates were then separated on SDS-PAGE and proteins were transferred onto a nitrocellulose transfer membrane (Whatman GmbH, Dassel, Germany). Polyclonal rabbit antibodies against iNOS (NB300-605, 1:500, Novus Biologicals, Ltd., Cambridge, UK) and arginase 1 (H-52, 1:500, Santa Cruz Biotechnology Inc., Heidelberg, Germany) were used for incubation O/N at 4°C. The exposure time of the secondary antibody (horseradish peroxidase-conjugated anti-rabbit IgG; 1:5,000 Santa Cruz Biotechnology Inc.) was 1 h at RT. Actin was detected using monoclonal anti-mouse actin antibody (ab6276, 1:10,000, abcam, Cambridge, UK) and a secondary horseradish peroxidase-conjugated antibody (anti-mouse IgG, ab6728, 1:2,000, abcam). Peroxidase-bound protein bands were visualized using the enhanced chemiluminescence method.

### Organotypic slice culture (OSC) preparation

OSCs were prepared as described by [45]. In brief, brains from 2 to 3 month old C57BL/6 mice were removed and immediately immersed in ice-cold Ringer solution [2.5 mM KCl (Merck KGaA, Darmstadt, Germany), 1 mM MgCl_2_ (Sigma-Aldrich), 260 mM d-Glucose (Applichem), 26 mM NaHCO_3_ (Merck KGaA), 1.25 mM NaH_2_PO_4_ (Merck KGaA), 2 mM pyruvic acid (PAA Laboratories), 3 mM myo-inositol (Sigma-Aldrich), 1 mM kynuric acid (Sigma-Aldrich), 2 mM CaCl_2_ (Carl Roth GmbH), pH 7.3]. The brains were embedded in 4% low melting agarose (Sigma-Aldrich). After polymerization agarose blocks were trimmed and glued onto the cutting table of a vibratome (VT1000, Leica, Heerbrugg, Switzerland). Brains were cut in coronal slices of 250 µm. Slices were then collected and stored in ice-cold Ringer solution before floating onto semi-porous membrane inserts (Millipore, 0.4 mm pore diameter) according to Stoppini et al. [46]. The slices were cultivated in wells of a 6-well plate at 37°C under 5% CO_2_ in a standard medium consisting of DMEM/Ham’s F12 (pH 7.3; PAA Laboratories), 24% normal horse serum (PAA Laboratories), 2% HEPES (PAA Laboratories), 0.1% gentamycin (PAA Laboratories) and additional 10 mM d-glucose (Sigma-Aldrich). Medium was changed every other day and viability of brain slices was assessed using PI staining.

### Implantation of tumor cells into OSCs

1 × 10^4^ U87-MG-RFP or GL261-RFP cells in a volume of 1 µl PBS were implanted with a Hamilton syringe into organotypic slice cultures 7 days after slice preparation. Starting 1 day after implantation, glioma growth and invasion were evaluated every other day using a MZ16 FA Stereo-Fluorescence Microscope (Leica, Wetzlar, Germany).

### Viral replication in OSCs

7 days after tumor cell implantation, the slices (5 slices/6-well) were infected with 5 × 10^6^ or 1 × 10^7^ plaque forming units (pfu) of virus diluted in 50 µl infection medium (DMEM/F12 + 2% FBS). Briefly, growth medium was replaced by 1 ml of infection medium and 10 µl virus solutions were pipetted onto each slice. The cultures were incubated for 3 h at 37°C. Then, 1 ml growth medium was added to the culture medium. Sections were harvested 24 and 72 h after infection. Following three freeze–thaw cycles serial dilutions of the lysates were titrated by standard plaque assay on CV-1 cells. All samples were measured in triplicate.

### Treatment and application of BV-2 cells onto OSCs

To stimulate BV-2 microglia before application onto the OSCs, cells were treated with 10 ng/ml rm-IFN-γ or 10 ng/ml rm-IL-4 (Immunotools GmbH) for 24 h. BV-2 cells were then administered directly onto the implanted OSCs in a volume of 2 µl PBS containing 1 × 10^5^ BV-2 microglial cells. Unstimulated BV-2 cells were used as controls.

### Staining of OSCs

24 and 72 h after infection OSCs were fixed with 4% paraformaldehyde (AppliChem) in PBS, pH 7.4, for 16 h at 4°C followed by three repeated washing steps for 15 min in PBS. The slice cultures were stored in PBS at 4°C. Sections were blocked for 1 h with 5% FBS in PBS supplemented with 0.3% Triton X-100. Primary antibodies against the glial fibrillary acidic protein (chicken anti-GFAP, ab4674, 1:100, abcam), specific for astrocytes, Iba-1 (rabbit anti-Iba-1, 1:100, Wako, Neuss, Germany) or FITC-coupled *Griffonia simplicifolia* Isolectin B4 (IB4) (1:50, Sigma-Aldrich), specific for microglia and anti-VACV antibody (rabbit anti-Vaccinia, ab35219, 1:100, abcam) were applied into the blocking solution.

The slices were incubated for 3 days at 4°C with orbital shaking. Then the slices were washed three times with PBS (10 min each) and incubated with specific fluorescence-labeled secondary antibodies (Cy2-donkey anti-rabbit 1:200, Cy5-donkey anti-chicken 1:200, both Dianova, Hamburg, Germany) in PBS for 4 h. The cell nuclei were stained subsequently with Hoechst 33342 (Sigma-Aldrich). The procedure was finalized with three washing steps before the tissue slices were mounted on glass slides and embedded in mounting medium. All control experiments were performed without primary antibodies. Images were taken under the MZ16 FA Stereo-Fluorescence microscope (Leica) equipped with a digital CCD camera (DC500, Leica) and at the Leica TCS SP2 AOBS confocal microscope equipped with an argon, helium neon and UV laser. Digital images (1,024 × 1,024 pixel RGB-color images) were processed with Photoshop CS2 (Adobe Systems, Mountain View, CA) and merged to yield pseudo-colored images.

### Intracranial tumor cell implantation and intratumoral/intracranial virus injection

Animal studies were performed in accordance with protocols approved by the Institutional Animal Care and Use Committee (IACUC) of Explora Biolabs (San Diego, CA, USA, study number GL13-05B and GL13-17, protocol number EB11-025) or the government of Unterfranken (Wuerzburg, Germany, protocol number AZ 55.2-2531.01-62/11). Four to five week old C57BL/6 or Foxn1 ^nu/nu^ mice (Harlan) were anesthetized by intraperitoneal injection of a ketamine (Ketavet, Pharmacia GmbH, Berlin, Germany) and xylazine (Xylavet, CP-Pharma GmbH, Burgdorf, Germany) cocktail and immobilized in a stereotactic apparatus (Stoelting Instruments). GL261 tumor cells (1x10^5^ cells in 2 µl PBS) were implanted over a 5-min period, at coordinates 1.0 mm anterior and 2.0 mm lateral to the bregma and 2.0 mm in depth using a Hamilton syringe. The incision was closed with surgical staples. Virus treatment was started 10 days after intracranial tumor cell implantation by a single intracranial/intratumoral injection (5 × 10^6^ pfu in 2 µl PBS) over a 5-min period of time. Mice without a tumor implantation just received the intracranial virus injection. Three mice per cohort were used in the study.

### Immunohistochemical analyses of mouse brains

For histological studies, brains were excised and snap-frozen in liquid nitrogen, followed by fixation in 4% paraformaldehyde/PBS pH 7.4 O/N at 4°C. Fixed brains were rinsed with PBS and embedded in 5% (w/v) low-melting agarose, followed by preparation of sections (100 µm) using a Leica VT1000S Vibratome. Tissue specimens were permeabilized and blocked in blocking solution for 1 h. The tissue sections were then labeled with the primary antibodies anti-Iba-1, anti-GFAP and anti-VACV for 12–15 h. Sections stained with IB4-FITC were incubated for an additional 24 h. After three washing steps in PBS, sections were incubated with secondary antibodies [Dylight 488-, Cy3- and Alexa Fluor 647-conjugated (Dianova)] and Hoechst 33342 for 5 h. All sections were rinsed with PBS. In a final step, the sections were mounted onto glass slides in Mowiol 4–88 (Sigma-Aldrich). The fluorescent-labeled preparations were examined as described above.

### Measurement of the fluorescence intensity of Iba-1 and GFAP in GL261 brain tumor sections

Measurement of the fluorescence intensity of Iba-1 and GFAP stained slices was performed on 100 µm agarose sections (three brain tumors per treatment conditions, in duplicate staining). Images were taken with identical camera settings. As described by Donat et al. [47]. RGB-images were converted into 8-bit gray scale with an intensity range from 0 to 255 with Photoshop CS5 (Adobe Systems, USA). The fluorescence intensity of Iba-1 and GFAP within the GL261 tumors represents the average brightness of staining related pixels and was measured with ImageJ. The tumor areas were determined with ImageJ. The fluorescence intensity was calculated as a ratio to the tumor area.

### Statistical analysis

To determine significance between two conditions or treatment groups, a 2-tailed *t* test with unequal variances was used (Excel 2010 for Windows). P-values were defined as follows *p < 0.05, **p < 0.01, ***p < 0.001. For calculating boxplot diagrams a template from Vertex42 LLC has been used.

## Results

### Replication of intratumorally injected VACV LIVP 1.1.1 was inhibited in orthotopic GL261 gliomas in mice

**Figure 1:**
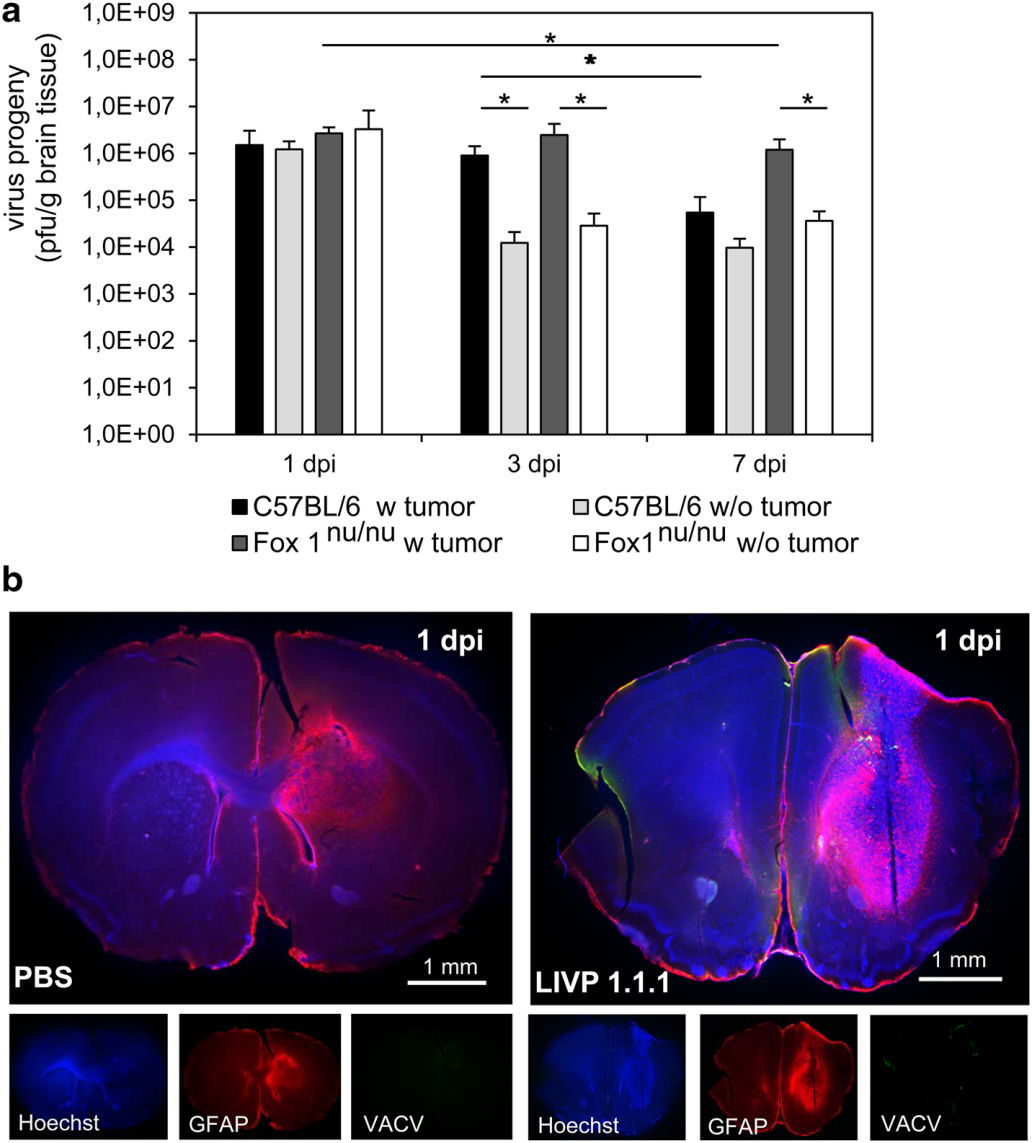
Injection of VACV into mouse brain tissues with and without implanted gliomas. **a** C57BL/6 and Foxn1^nu/nu^ mice with (w) and without (w/o) GL261 brain tumors were infected with 5 × 10^6^ pfu LIVP 1.1.1 (i.t). For analysis of viral titers, brains were excised 1, 3 and 7 days after virus injection and analyzed by standard plaque assay [n = 4/5 per group (w) and n = 3 per group (w/o) tumor]. All samples were measured in duplicate (data are mean values ± SD; t test *p < 0.05). **b** Immunohistochemical staining for astrocytes (anti-GFAP, *red*) and VACV (anti-VACV, *green*) 1 dpi in GL261 implanted tumors. Shown are representative examples.

To analyze VACV progeny in orthotopic GL261 gliomas in vivo, two different mouse strains, immunocompetent C57BL/6 wild-type mice and immunodeficient athymic Foxn1^nu/nu^ mice, were injected intratumorally with 5 × 10^6^ pfu/brain LIVP 1.1.1. Mice of both strains without GL261 gliomas (w/o) were used as controls (Figure 1a). We found that the virus titers were reduced in C57BL/6 wild-type mice with and without GL261 tumors, from initially injected 5 × 10^6^ pfu/brain to 1 × 10^6^ pfu/g brain tissues 1 day post infection (dpi). The titer further decreased to 5 × 10^4^ and 1 × 10^4^ pfu/g brain tissues at 7 dpi, respectively. Strikingly, similar results were obtained in immunodeficient Foxn1^nu/nu^ mice. Only very few LIVP 1.1.1 viral particles were detectable by immunohistochemistry within tumors at 1 dpi (Figure 1b). This data implicates that VACV LIVP 1.1.1 did not replicate efficiently in orthotopic GL261 gliomas nor did it in healthy brains of immunocompetent and immunodeficient mice. Only a very minor part of the viral inoculum remained viable within the brain for the 7 day time period.

### Intratumoral injection of VACV into orthotopic GL261 brain tumors did not alter the intratumoral amounts and distribution of microglia and astrocytes in C57BL/6 mice

**Figure 2:**
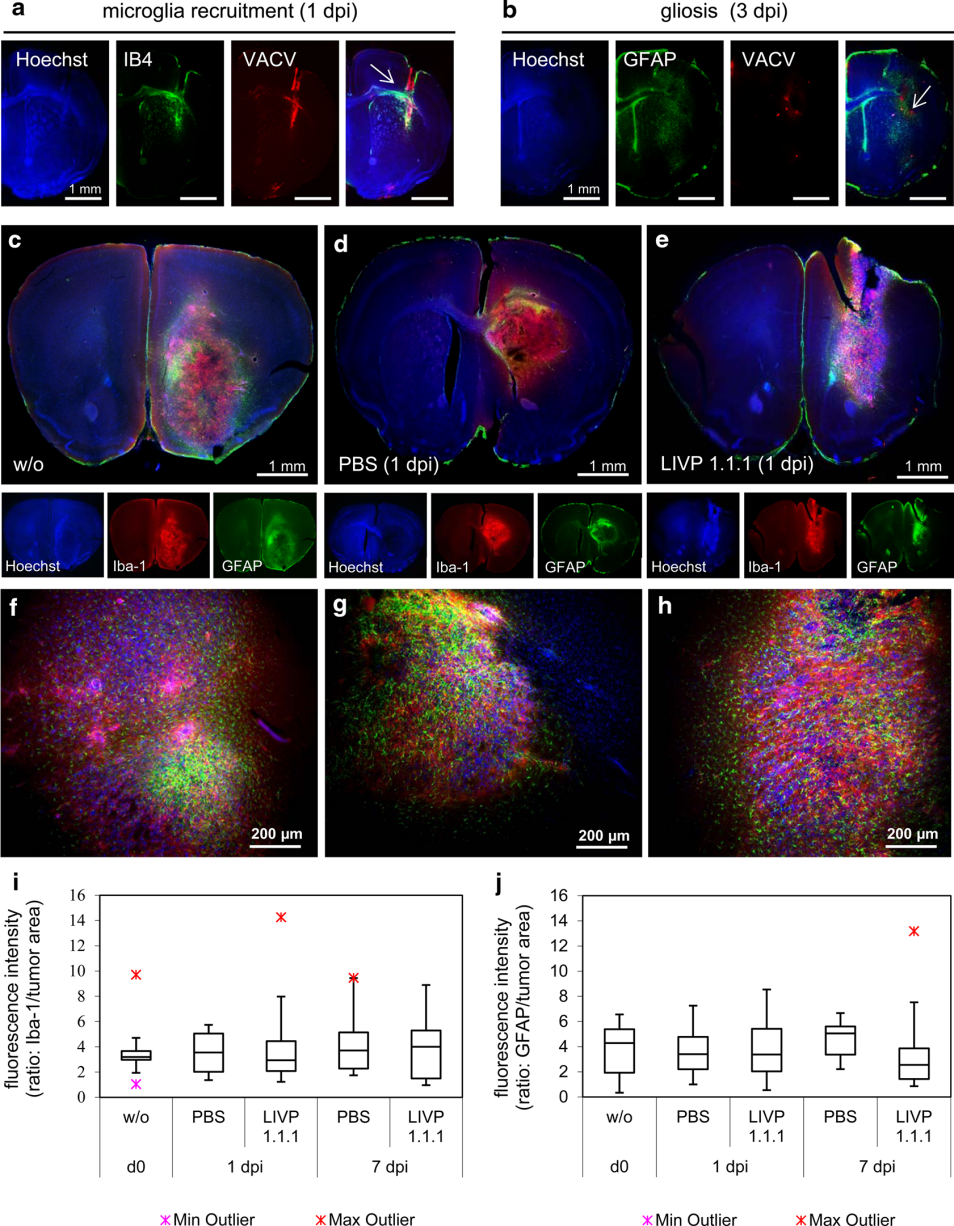
Recruitment and infiltration of microglia and astrocytes into murine gliomas. **a**, **b** Recruitment of microglia and astrocytes 1 and 3 days after intracranial injection of GLV-2b372 into naïve Foxn1^nu/nu^ mice stained with Hoechst (*blue*) to detect cell nuclei, IB4 (*green*) to stain microglia and GFAP to stain astrocytes (*green*). Virus was detected by expression of TurboFP635 (*red*). The VACV injection sites are marked by *white arrows* in the overlays. Immunohistochemical staining and analysis of GL261 gliomas in C57BL/6 mice untreated (w/o; **c**, **f**), injected intratumorally with PBS (**d**, **g**) or LIVP 1.1.1 (5 × 10^6^ pfu) (**e**, **h**) at 1 dpi. Sections were stained for cell nuclei (*blue*), microglia (Iba-1^+^, *red*) and astrocytes (GFAP^+^, *green*). **i**, **j** Quantitative analysis of microglia and astrocytes 1 and 7 dpi (n = 3; analyzed in duplicate). Fluorescence intensity and tumor area were measured with ImageJ and calculated as ratio: Iba-1 or GFAP/tumor area. For box plot diagrams a template from Vertex42 LLC has been used.

In a further step, the virus injection site in athymic Foxn1 ^nu/nu^ mice without brain tumors was analyzed by immunohistochemical stainings of brain sections 1 and 3 days post infection (dpi). For infection 5 × 10^6^ pfu of the red fluorescent VACV strain GLV-2b372 was injected intracranially. A strong infiltration of immune cells to the injection site could be detected (Figure 2a, b): microglial cells stained with the microglial marker Iba-1 were already recruited 1 day after virus injection, whereas astrocytes stained with astrocytic marker GFAP were present after 3 days. VACV replication detected by expression of TurboFP635 was visible at the injection site (Figure 2a, b) but did not spread further through tissues.

Next, we set out to analyze the effects of VACV injection on these particular cell types under pathological conditions in wild-type mice with GL261 gliomas. The recruitment of both immune cell types was investigated in non-, mock-, and LIVP 1.1.1-infected tumors. Immunohistochemical analysis of C57BL/6 mouse brains 1 day post intratumoral infection with LIVP 1.1.1 (Figure 2e, h) or PBS as control (Figure 2d, g) revealed a strong infiltration of the tumor with microglia (Iba-1^+^) and astrocytes (GFAP^+^) at both treatment conditions. GL261 cells were tested negative for GFAP (data not shown). Surprisingly, tumors without injection showed a similar infiltration pattern (Figure 2c, f). Iba-1^+^ microglial cells were distributed homogenously throughout the tumor (center and rim), whereas GFAP^+^ astrocytes were located mainly at the periphery of the tumors (Figure 2c–e). However, quantitative analysis revealed no difference in the amount of Iba-1^+^cells and GFAP^+^ cells in untreated tumors compared to mock or virus injected tumors 1 and 7 dpi (Figure 2i, j). Taken together, these findings indicate that the presence of microglia and astrocytes in GL261 gliomas is the consequence of the growing tumor [12, 18–20] and not caused by VACV infection.

### In contrast to orthotopic GL261 gliomas, VACV replication was high in glioma tumor cells, very low in BV-2 microglial cells, and absent in IMA2.1 astrocytes in cell culture

**Figure 3:**
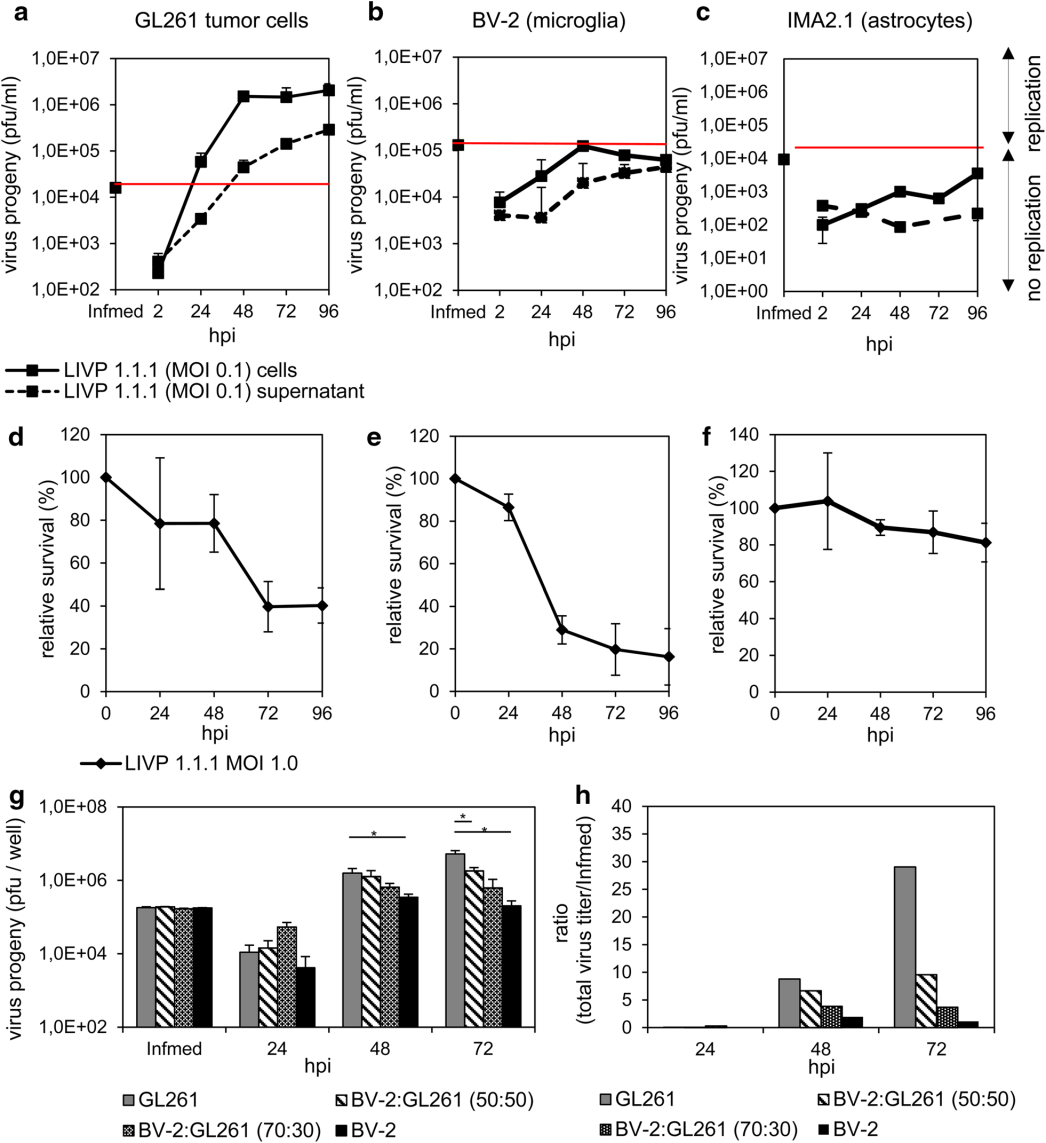
Analysis of VACV infections in murine GL261, microglial BV-2 and astrocytic IMA2.1 cells in cell culture. **a**–**c** Viral replication in GL261, BV-2 and IMA2.1 cells infected with LIVP 1.1.1 at an MOI of 0.1 was analyzed by standard plaque assay. The *red line* separates active replication from no replication. **d**–**f** MTT assay was performed to detect the percentage of surviving cells after infection with LIVP 1.1.1 (MOI 1.0). **g** BV-2 and GL261 cells were cultured as direct co-cultures for 24 h in various concentrations. For standard plaque assay cells were infected at an MOI of 0.1 in triplicate. **h** Viral titers were related to the infection medium for a better comparison and illustrated as ratio (total virus titer/infection medium). Experiments were repeated in independent experiments. Two-sided t test with unequal variances was used for statistics (*p < 0.05).

One explanation of reduced VACV replication in the mouse brain could be the preferential uptake of viral particles into microglia or astrocytes. We tested this hypothesis in cell culture, using two specific cell lines, BV-2 and IMA2.1, with similar growth characteristics as primary cells. To determine LIVP 1.1.1 virus progeny in these cell lines in vitro, cells were infected at an MOI of 0.1 followed by standard plaque assay performed at different time points. Data was compared with infection of GL261 glioma cells under the same culture conditions (Figure 3a–c). We confirmed that LIVP 1.1.1 replicated efficiently in GL261 cells, determined by increasing cell-associated virus titers from 2 to 48 h post infection (hpi) and a plateau phase reached at 48 hpi at a virus dose of 1.5 × 10^6^ ± 2.1 × 10^5^ pfu/ml. Already, 24 hpi virus titers were higher than the infection medium (1.6 × 10^4^ ± 2.3 × 10^3^ pfu/ml). Virus titers in the supernatant steadily increased over time, reaching a maximum at 96 hpi (2.9 × 10^5^ ± 5.1 × 10^4^ pfu/ml) (Figure 3a). In contrast, the virus titer in BV-2 cells remained slightly below the infection medium at all times that were analyzed except for the 48 hpi time point (Figure 3b). Surprisingly, in IMA2.1 cells, the virus titer was far below the infection medium during the whole time course of the experiment (Figure 3c). No virus spreading occurred as indicated by the complete lack of viral particles in the supernatant of BV-2 and IMA2.1 infected cells. Cell viability after virus infection was examined by MTT assay at an MOI of 1.0. The analysis showed that GL261 and BV-2 cells were lysed upon infection with LIVP 1.1.1, whereas IMA2.1 cells were intact with a slight decrease in the amount of living cells (Figure 3d–f).

To analyze the competitive factors affecting virus replication we set up direct co-cultures of BV-2 and GL261 cells in various concentrations for 24 h. For standard plaque assays cultures were infected at an MOI of 0.1 in triplicates (Figure 3g–h). Virus replication was significantly higher when GL261 cells were cultured alone than with BV-2 cells (50:50) 72 hpi. These effects can also be illustrated as ratio of the analyzed virus titers in culture and the infection medium (Figure 3h). In conclusion, these experiments showed that the more BV-2 cells are present in the co-culture the less viral particles are detected over time. This finding may indicate that BV-2 microglia may compete for virus uptake in culture.

### Apoptosis and necrosis studies with BV-2 and GL261 cells

VACV is known to induce either apoptosis or necrosis in different cell lines. Dependent on the infected cell type, apoptosis can be associated with reduced virus infection or replication [48]. Since replication analysis of LIVP 1.1.1 in BV-2 and GL261 cells revealed a diverse efficacy of viral replication, but virus-mediated cell death was observed in both cell types, we investigated different mechanisms of cell death, i.e. apoptosis and necrosis, in these cell lines.

**Figure 4:**
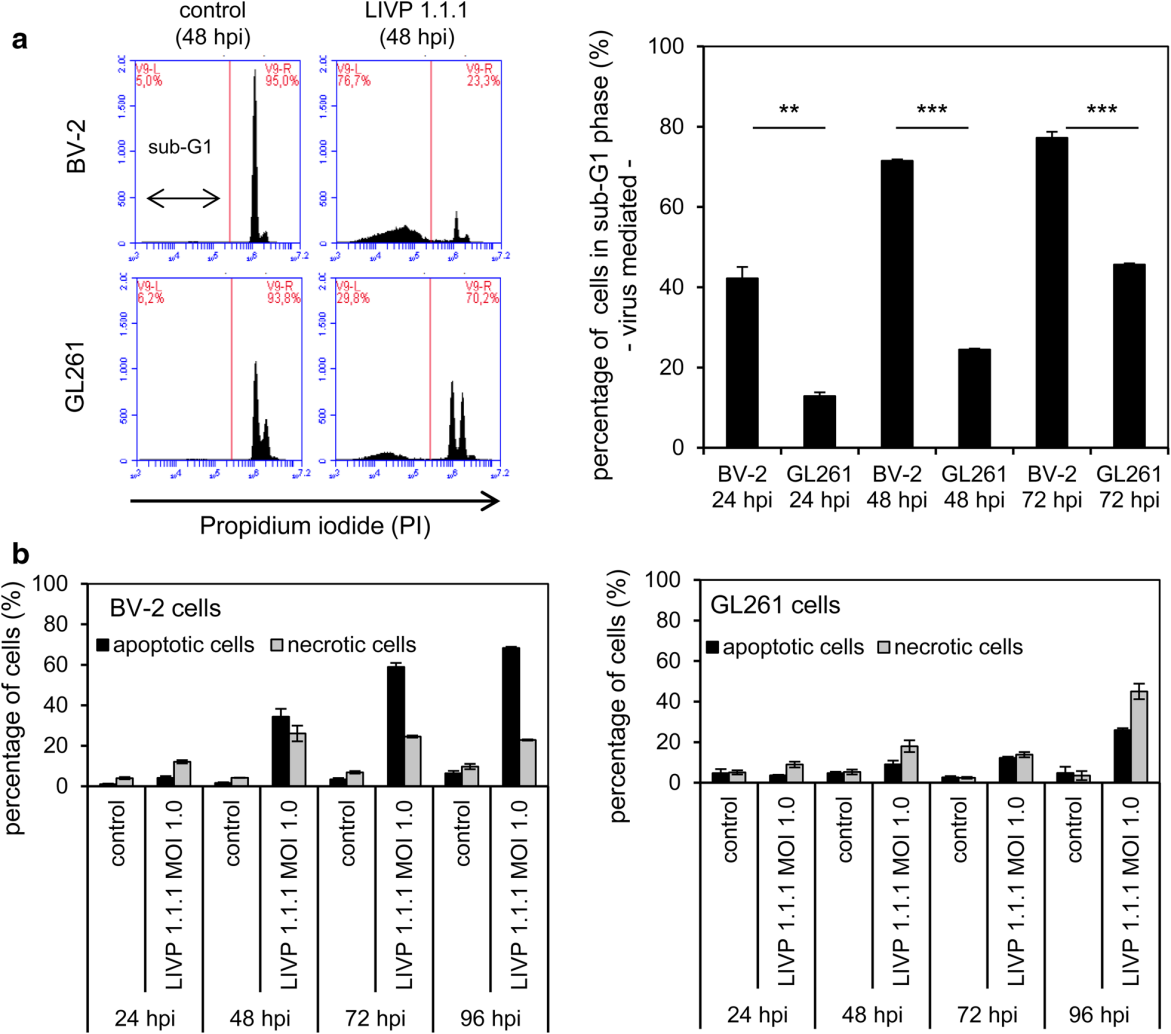
FACS analyses of GL261 and BV-2 cells. **a** Cells that have lost part of their DNA due to DNA fragmentation in apoptosis are represented in sub-G1 peaks on DNA histograms. The percentage of BV-2 or GL261 cells in sub-G1 phase 24, 48 or 72 h after viral infection (MOI 0.5) are shown in the bar chart. **b** The percentages of PI-dim (apoptotic) and PI-bright (necrotic) cells in controls and LIVP 1.1.1-infected samples (MOI 1.0) are shown for BV-2 and GL261 cells. The experiment was performed in triplicate and repeated in an independent experiment.

To quantify late apoptosis, the percentage of cells in sub-G1 phase was analyzed by flow cytometry. The percentages of mock-infected and VACV-infected cells in sub-G1 phase were compared at 24, 48, and 72 hpi (Figure 4a). We found that the percentage of cells in sub-G1 phase was higher in BV-2 cells than in GL261 cells at all times. 40% of BV-2 cells were in sub-G1 phase after 24 h and this amount of cells increased over time to 70% after 48 h and to 80% after 72 h. On the other hand, at 24 hpi the amount of GL261 cells in sub-G1 phase was around 10% and increased to 45% after 72 h (Figure 4a). Additionally, distribution of PI-positive cells in PI-dim (apoptotic) and PI-bright (necrotic) cells [43, 44] revealed higher numbers of apoptotic cells in the VACV-infected BV-2 sample compared to infected GL261 cells at all time points (Figure 4b). Our data indicates that VACV infection activates different cell death pathways and mechanisms in microglial BV-2 cells in contrast to GL261 tumor cells.

### LPS and IFN-γ treatment in microglial BV-2 cells further reduced virus progeny in vitro

**Figure 5:**
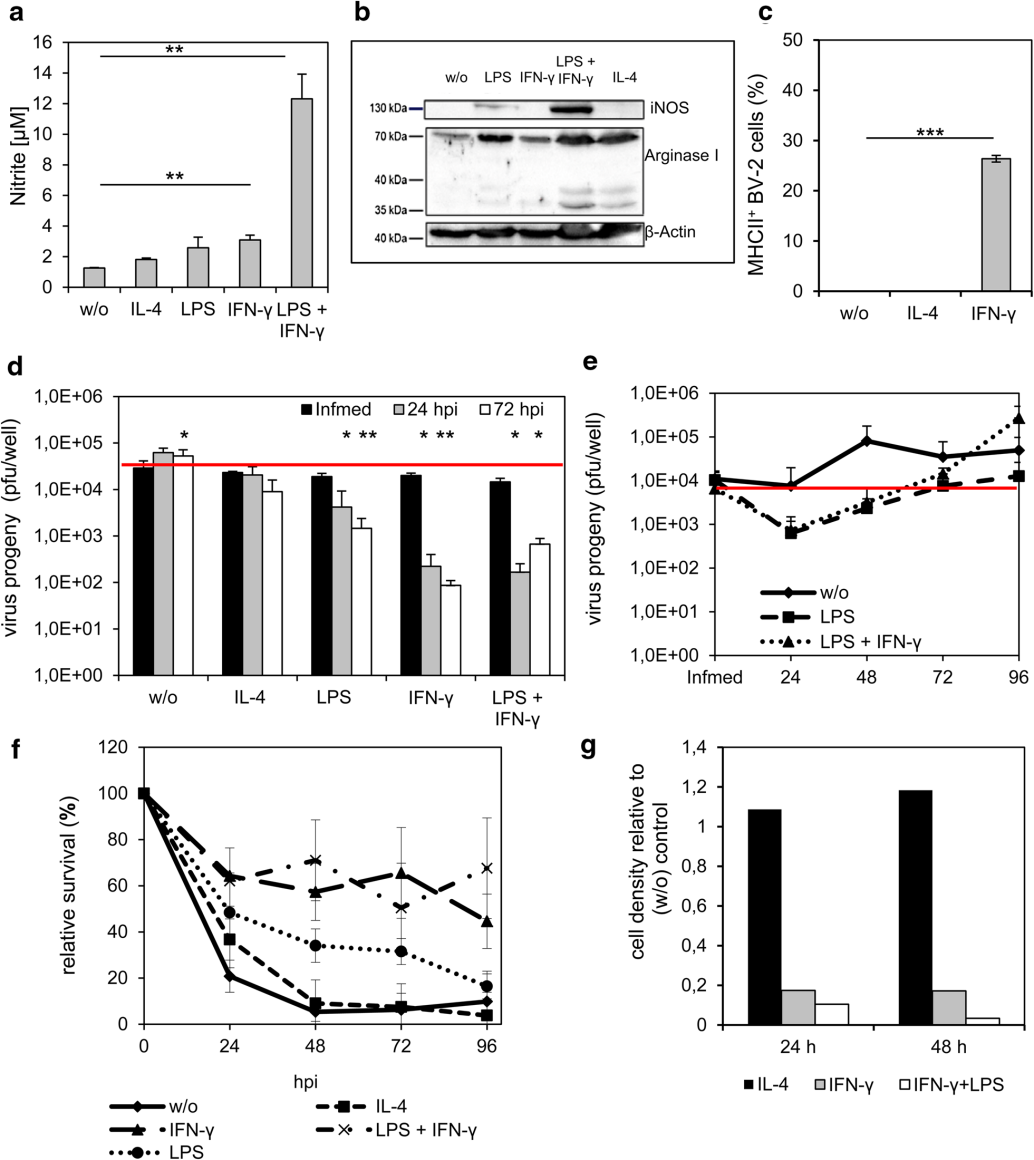
Impaired amount of virus progeny after stimulation of BV-2 cells with IFN-γ or IFN-γ and LPS. BV-2 cells were stimulated with LPS (10 µg/ml), IFN-γ, (10 ng/ml), LPS + IFN-γ or IL-4 (10 ng/ml) for 24 h in medium with 2% FBS. Polarization of cells was analyzed by Griess assay (**a**), Western blot (**b**) and by detection of the percentage of MHCII^+^ cells by FACS analysis (**c**). The amount of virus progeny (pfu/ml) was analyzed by standard plaque assay in the presence of stimulating factors and in normal growth medium in cells and supernatants (**d**). Statistical analysis was performed related to infection medium. To analyze the recovery of viral replication, normal growth medium was applied after infection of pre-stimulated cells. Standard plaque assay was performed to determine viral progeny in cells and supernatant after 24, 48, 72 and 96 hpi (**e**). To analyze the relative survival of the cells in the presence of stimulating factors a MTT assay was performed (**f**). The cellular density of stimulated cells relative to unstimulated (w/o) cells was detected via optical density measurement after 24 and 48 h of cultivation (**g**). Two-sided t test with unequal variances was used for statistics *p < 0.05, **p < 0.01, ***p < 0.001. Active replication was defined as virus titer above the virus titer of the infection medium (*red line*). All experiments were performed in triplicate and repeated in an independent experiment.

Like peripheral macrophages also microglia take on either a classically activated M1 phenotype or an alternatively activated M2 phenotype [24]. We therefore set out to analyze both M1 and M2 phenotypes of BV-2 microglia for their ability to take up and support LIVP 1.1.1 virus replication. The two different phenotypes M1 and M2 are dependent on exogenous addition of certain stimuli. Such stimuli are IL-4 for induction of the M2 phenotype, and IFN-γ or LPS or a combination of both for induction of the M1 phenotype [49]. LPS is known to induce nitric oxide (NO) production in BV-2 cells, a marker for the M1 phenotype [40, 50]. As shown in Figure 5a a low NO production indicated by the measurement of nitrite via Griess assay, was induced by treating BV-2 cells with IFN-γ or LPS alone. However, when cells were treated with IFN-γ and LPS in combination, NO production was at the highest level, indirectly measured by the amount of nitrite (12.31 ± 1.61 µM). Western Blot analysis of total protein amounts extracted from treated BV-2 microglial cells indicated that the increase in NO production by IFN-γ and LPS resulted in an up-regulation of the inducible isoform of nitric oxide synthase (iNOS) (Figure 5b). In addition, treatment of BV-2 cells with IL-4 or LPS both increased the expression of arginase 1, a marker for the M2 phenotype. Treatment of BV-2 cells with IFN-γ led to a reduction of arginase 1 expression. Furthermore, treatment of BV-2 cells with IFN-γ for 24 h resulted in high-level expression of MHCII molecules, an indicator for the M1 phenotype, whereas MHCII expression was not detectable in unstimulated or IL-4 stimulated cells detected by FACS analysis (Figure 5c). Cells pre-incubated with LPS, IFN-γ, or LPS and IFN-γ and infected with LIVP 1.1.1 revealed a reduced amount of viral progeny 24 and 72 hpi (Figure 5d). In contrast, there was no difference between IL-4 or unstimulated cells. Interestingly, viral replication could be restored after medium change. We detected a continuous increase of the virus titer in cells pre-incubated with either LPS or LPS and IFN-γ when cells were cultured in normal growth medium after virus infection. The virus titer was higher than the infection medium at 72 and 96 hpi, respectively (Figure 5e). MTT assay revealed that around 50% of cells stimulated with IFN-γ or LPS and IFN-γ were alive 96 hpi, ~20% of surviving cells were detected in the LPS treated samples and less than 10% of surviving cells were detected in the IL-4 and unstimulated samples (Figure 5f). Furthermore, BV-2 cells which were stimulated with IFN-γ and/or LPS proliferated to a lesser extent than unstimulated or IL-4 stimulated cells as indicated by the cellular density at 24 and 48 h which was calculated relative to unstimulated cells. (Figure 5g). Based on virus replication and virus-mediated cell death, these results strongly indicate preferred virus infection of cells of the M2 phenotype or unstimulated form of BV-2 cells in comparison to the M1 phenotype.

### Application of LPS and IFN-γ negatively affected viral particle production in IMA2.1 astrocytic cells

**Figure 6:**
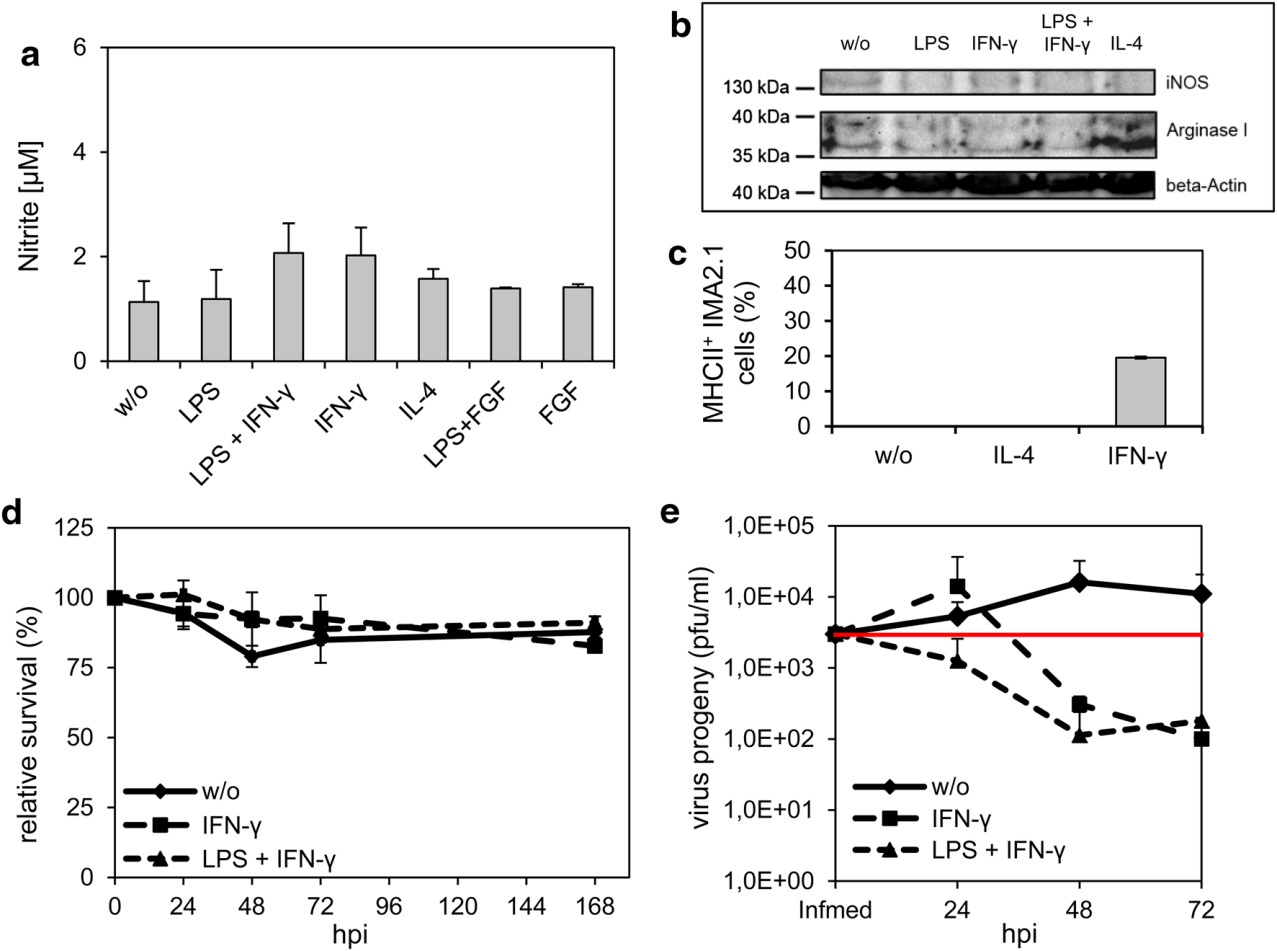
Stimulation of IMA 2.1 cells had no effect on relative survival after viral infection. IMA 2.1 cells were stimulated with LPS (10 µg/ml), IFN-γ, (10 ng/ml) LPS + IFN-γ, IL-4 (10 ng/ml), FGF (5 ng/ml) or FGF + LPS for 24 h in medium with 2% FBS. Polarization of cells was analyzed by Griess assay (**a**), Western blot (**b**) and FACS analysis by detection of MHCII^+^ cells (**c**). **d** MTT assay was performed to detect the relative percentage of VACV-mediated cell death in the presence of stimulating factors. **e** Viral replication was analyzed by standard plaque assay in the presence of stimulating factors. All experiments were performed in triplicate.

Polarization towards the M1 or M2 phenotypes is also known to occur in activated astrocytes [29]. The cell line IMA2.1 is known to respond similarly to stimuli like primary astrocytes [37]. We tested the ability of different pro-inflammatory cytokines (IL-4 and IFN-γ) and LPS to induce NO production in IMA2.1 cells. We also tested the effect of bFGF on IMA2.1 cells since bFGF like IL-4 is known to enhance the proliferation of astrocytes [51]. We have found that there was no increase in NO levels after treatment with different stimulating factors shown as measurement of nitrite in Figure 6a. In addition, iNOS expression levels were not detectable via Western blot. Only arginase 1 was detectable after IL-4 treatment (Figure 6b). Surprisingly, FACS analyses revealed increased MHCII expression in IMA2.1 cell cultures after stimulation with IFN-γ for 24 h as marker for the M1 phenotype (Figure 6c).

There was no effect on virus-mediated cell death under any of the treatment conditions (Figure 6d). Infection of cells treated with IFN-γ or LPS and IFN-γ resulted in a further reduction of viral particles compared to unstimulated cells (Figure 6e). Furthermore, the amount of viral particles in cell cultures remained below the levels of the infection medium under all treatment conditions.

### LIVP 1.1.1 did not replicate efficiently in GL261 tumor spheres in organotypic slice cultures

**Figure 7:**
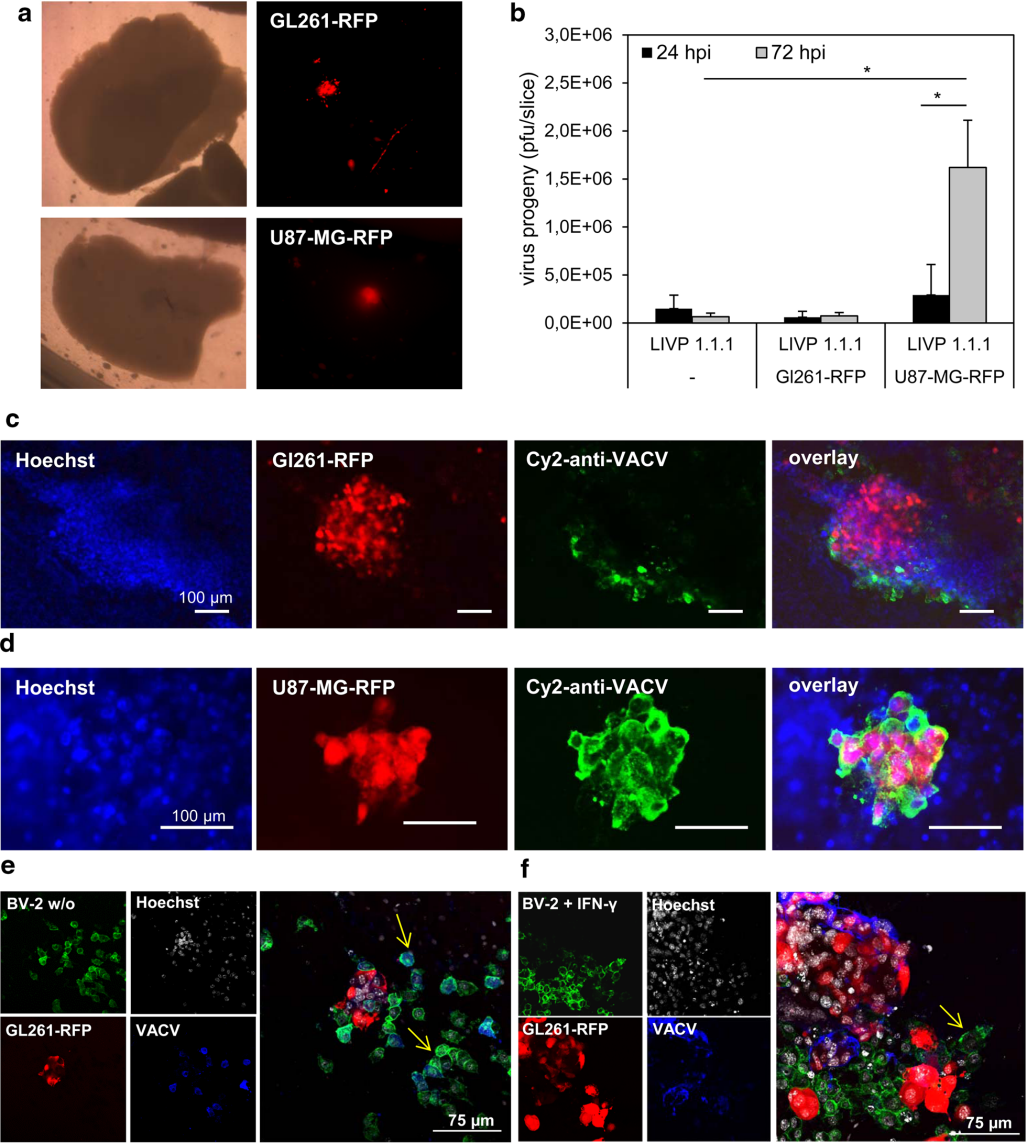
Analysis of VACV infection in organotypic slice cultures with tumor cell implants. **a** Organotypic slice cultures (OSCs) from adult mice were established and implanted with 1 × 10^4^ Gl261-RFP or U87-MG-RFP cells. 7 days after implantation, OSCs were infected with 5 × 10^6^ pfu LIVP 1.1.1/well. **b** Standard plaque assay was performed 24 and 72 h after infection. Two-sided t test with unequal variances was used for statistics *p < 0.05. 72 h after infection OSCs implanted with GL261-RFP (**c**) and U87-MG-RFP (**d**) cells were stained with Hoechst and anti-VACV antibody to visualize infected tumor spheres. **e**, **f** IFN-γ-stimulated or unstimulated BV-2 cells were applied directly onto the OSCs implanted with GL261 cells. 24 h later OSCs were infected with LIVP 1.1.1 and stained after additional 24 h with FITC-IB4, Hoechst and anti-VACV antibody. *Yellow arrows* indicate exemplarily infected or non-infected BV-2 microglial cells.

We established organotypic slice cultures (OSCs) from 2 to 3 month old C57BL/6 mice, the same age as the mice that received intracranial injections. The murine GL261 glioma cell line can be viewed as syngeneic non-responder tumor model [52] and the human U87-MG glioblastoma cell line, in contrast, as the responder model [53]. OSCs were implanted with either GL261-RFP or U87-MG-RFP cells and the tumors were allowed to establish for 7 days. The OSCs containing the tumors were infected with 5x10^6^ pfu LIVP 1.1.1 by adding VACV directly onto the tumor spheres (Figure 7a). Viral plaque assay of OSCs with GL261-RFP tumors revealed no difference of the virus titer between 24 and 72 h. In contrast, U87-MG-RFP tumors showed a tenfold increase of VACV LIVP 1.1.1 progeny after 72 h (Figure 7b). Immunohistochemical staining of tumor spheres (Figure 7c) confirmed that VACV is able to infect GL261 tumors expressing RFP but viral particles were only detectable at the periphery of the tumor. In contrast, OSCs with U87-MG tumors expressing RFP were completely infected 72 h after addition of the virus onto the slices (Figure 7d). The same results were observed in OSCs derived from an immunodeficient mouse brain (data not shown). Here the results obtained from intracranial implantation and infection experiments were also confirmed in OSC tumors ex vivo.

### Microglia could be protected from infection with VACV LIVP 1.1.1 in OSCs

With OSCs we further attempted to determine the role of phagocytic microglia in mouse brain tumors. We performed exogenous application of BV-2 microglia to the OSCs which were treated with different cytokines in advance. GL261 cells were implanted into OSCs and allowed to establish for 7 days. In a second step microglial BV-2 cells either non-stimulated or stimulated for 24 h with IFN-γ or IL-4 were added onto OSCs. One day after addition of BV-2 microglial cells, OSCs were infected with VACV for 24 h. BV-2 microglial cells were stained with FITC-IB4. Representative images of immunohistochemical stainings showed that unstimulated BV-2 cells located around the tumor cells were infected with LIVP 1.1.1, whereas the GL261 tumor cells were only partially infected (Figure 7e). Studies of BV-2 cells stimulated with IL-4 prior to application showed similar results (data not shown). Interestingly, BV-2 cells stimulated with IFN-γ for 24 h prior to application were not infected, however GL261 tumor cells in contrast were well infected (Figure 7f). From these promising preliminary results we concluded that OSCs are a suitable method to model ex vivo interactions of conditioned immune cells, intracellular pathogens and tumor cells for the understanding of possible interactions in the tumor microenvironment.

## Discussion

In this study, we examined how glial cells and the tumor microenvironment affected the efficacy of oncolytic VACV LIVP 1.1.1 infection in intracranial GL261 tumor models and in cell culture systems. It is common knowledge that glioma cells release factors for active attraction of immune cells at injury- and tumor sites [21, 54, 55]. Here we found that non-infected GL261 tumors at an early stage are heavily infiltrated by microglia and astrocytes, most likely recruited by the tumor cell implantation. Interestingly, the amounts of microglia and astrocytes were not significantly different in PBS- and VACV-injected tumors at day 1 and 7. It was observed that amounts of microglia were increased in proportion with tumor growth. We concluded therefore that preexisting infiltration in tumors and not the VACV injection caused the extensive recruitment. Further results showed that LIVP 1.1.1 was neither able to replicate efficiently in the orthotopic GL261 tumors nor in the healthy brains of immunocompetent as well as immunodeficient mice.

No significant differences between virus titers of immunodeficient and immunocompetent mice with orthotopic GL261 gliomas were detected. This finding may be explained by the fact that the brain in contrast to the periphery is defined as an “immune privileged” or “immune specialized” organ [56–59]. The differences between the immune system in the brain and the periphery include but are not limited to a reduced number of antigen-presenting cells in the brain tissues, only few and activated lymphocytes that can enter the brain tissues, no traditional lymphatic system present in the brain, and finally engrafted tissues in the CNS are slower rejected than transplants somewhere else in the body [56–59].

In a separate study we showed, that the VACV titer in subcutaneous GL261 tumors in C57BL/6 mice was dependent on endogenous IFN-γ levels within the tumor microenvironment [52]. Due to the constitution of the intracranial GL261 tumors possible explanations for the reduced viral replication might be in addition the uptake of viral particles into microglia and into astrocytes. This hypothesis was further analyzed in cell culture studies. For this experiment, the well-established murine microglial cell line BV-2 was used. We showed that BV-2 cells took up LIVP 1.1.1 but the amount of virus progeny was very low in comparison to GL261 glioma tumor cells. Infection of BV-2 microglial cells with LIVP 1.1.1 resulted in cell lysis in cell culture and we confirmed that VACV LIVP 1.1.1 induced apoptosis in murine BV-2 cells. However, more of the tumor cells exhibited a necrotic state after infection. Liskova et al. showed that VACV can induce either apoptosis or necrosis in different cell types [48]. Humlová and colleagues reported that VACV infection in fact induces apoptosis in J774 murine macrophages [60]. We argued that after virus infection microglial cells become apoptotic, and therefore no further viral spreading may occur.

We also found that the astrocytic cell line IMA2.1 was infected by LIVP 1.1.1 as determined by standard plaque assays. However, the replication was abortive. Furthermore, the lack of replication did not yield cell lysis. Taken together, as both BV-2 microglia and IMA2.1 astrocytes did not support active viral replication we concluded that both cell types may function as cell traps for VACV.

In gliomas the interplay with myeloid cells in the tumor microenvironment is a decisive factor [18]. Glioma cells secrete factors such as IL-10, IL-4, IL-6, M-CSF, TGF-β, and prostaglandin E2 that suppress the immune functions of microglia when these cells are located inside the tumor [21, 54]. These cytokines are known to promote a shift to M2 phenotypes and at the same time to suppress the M1 phenotype. Gliomas also secrete factors which lead to polarization of the M1 phenotype in microglia/macrophages. There is an intense controversy about the amounts of M1 and M2 phenotypes inside the tumor mass [17, 21, 22]. For example, Glass and Synowitz observed that tumor-associated macrophages in gliomas have an aberrant immune type which shares both M1 and M2 features [18]. In addition, findings concerning VACV infection of immune cells e.g. macrophages are controversially discussed in literature. To name only two examples: Broder et al. [61] mentioned that human primary macrophages do not support productive infection of VACV which is consistent with results from primary mouse and rabbit macrophages. In a study of Byrd et al. [62] it was demonstrated that primary human monocyte derived macrophages (MDMs) with either M1- or M2-phenotype do support VACV replication in contrast to AB-serum-derived MDMs.

In the present study, the M1 and M2 phenotypes of BV-2 microglia and IMA2.1 astrocytes were analyzed after infection with VACV LIVP 1.1.1. Table 1 summarizes the polarization experiments of both cell lines BV-2 and IMA 2.1 based on VACV replication and VACV-mediated cell death. The use of LPS or IFN-γ alone, LPS and IFN-γ in combination, all of which turn the cells into M1 phenotypes, resulted in a reduction of viral particles in BV-2 cells. LPS and IFN-γ together had cumulative effects. In unstimulated cells viral particles were detectable after 72 h but replication efficacy did not reach the initial infection medium. Treatment with IL-4 showed similar results when compared to unstimulated BV-2 cells.

**Table 1:** Summary of treatment conditions on BV-2 and IMA2.1 cells and their effects on VACV infection

Treatment condition	BV-2	IMA2.1	BV-2	IMA2.1
	Virus progeny^a^		VACV-mediated cell death^b^	
M2 phenotype				
w/o	+	0	+	−
IL-4	+	nt	+	−
M1 phenotype				
IFN-γ	−	−	−	−
IFN-γ + LPS	−	−	−	−
LPS	−	−	−	−

*nt* not tested, *w*/*o* without stimulation.

^a^Virus progeny (24 and 72 hpi) related to the infection medium was determined by standard plaque assays. [+] indicates an increase of the virus titer from the infection medium, [0] virus titer at the same level as the infection medium, [−] virus titer below the infection medium.

^b^VACV-mediated cell death was related to uninfected controls (24–72 hpi) and measured by MTT cell survival assays. [+] more than 50% of cells were killed by LIVP 1.1.1 [−] less than 50% of cells were killed by LIVP 1.1.1.

In astrocytic IMA2.1 cells VACV infection was found to be independent from the status of polarization. We found that VACV LIVP 1.1.1 did not replicate in IMA2.1 cells. Therefore we concluded that astrocytes restrict the oncolytic virus therapy in the mouse brain by not allowing VACV replication to occur.

Immunohistochemical analysis of gliomas treated with different classes of oncolytic viruses such as HSV or adenovirus have shown intratumoral elimination of over 80% of viral particles shortly after delivery. The elimination was shown to be associated with up-regulation and infiltration of cells of monocytic lineage both in immunocompetent and in immunodeficient tumor models [63, 64], confirming the findings with VACV LIVP 1.1.1 strain.

OSCs represent an excellent experimental linkage between cell cultures and live animal experiments, where anatomical, morphological, and cellular function of specific brain regions can be maintained and reconstructed. The main advantage of OSCs is that factors such as cell–cell and cell–matrix interactions affecting viral entry or replication are preserved in the intact three-dimensional milieu of the brain slice tumors [11]. We showed that LIVP 1.1.1 VACV infection resulted in no virus replication in GL261-RFP tumor spheres, and in contrast, very high replication in U87-MG-RFP tumor spheres. The U87-MG cell line is a well-characterized cell line that originated from an astrocytic tumor with necrosis [65]. It was previously shown that this cell line infiltrates brain slice cultures in an in vivo-like manner [11, 66, 67].

In addition, cell culture data and the results from OSC experiments showed evidence that BV-2 cells were infected first by LIVP 1.1.1 when BV-2 cells and GL261 cells were co-cultured or in a close microenvironment. Such infection was dependent on the polarization of the cells, e.g. stimulation with IFN-γ prevented BV-2 cells from infection with LIVP 1.1.1. IFN-γ mediated antiviral effects and mechanisms are reviewed in detail by Schroder et al. [68] and Chesler et al. [69].

Since microglial cells respond first to injury or to infection and also make up a significant part of the tumor mass, we concluded that they were one actual target for VACV uptake and infection. We propose that possible therapeutic approaches could be directed towards the modification of glioma-associated microglia. Successful VACV infection of gliomas could be altered by pre-stimulation of microglial cells so that, in consequence, tumor cells would be infected in the first instance. However, it is important to note that this study is based on cell lines that may not reflect exactly the behavior of primary cells, which needs to be shown in future studies.

As an outlook we assume that biopsies of individual cancer patients for ex vivo infection on OSCs could be a beneficial test system before starting a particular oncolytic virotherapy.

## Conclusions

The present study demonstrated that orthotopic GL261 gliomas in C57BL/6 and FoxN1^nu/nu^ mice were “non-responder” models for oncolytic virotherapy with VACV LIVP 1.1.1. By searching for explanations for this finding we detected that microglia and astrocytes were recruited to the tumor sites independent of intratumoral virus infection. In cell culture we could clearly show that microglia and astrocytes, dependent on their polarization into M1/M2 phenotypes, may preferentially clear viral particles by uptake immediately after delivery and therefore not allowing efficient virus infection. Based on our cell culture findings and OSC studies we assume that microglia compete for virus uptake with glioma cells. Reasons for reduced virus titers could be due to different cell death pathways (apoptosis vs. necrosis) activated by VACV infection in microglial BV-2 cells in contrast to GL261 tumor cells. Taken together our findings indicate that brain glia cells need to be taken into account for future therapy development.

## Abbreviations

bFGF: basic fibroblast growth factor
FBS: fetal bovine serum
dpi: days post infection
GBM: glioblastoma multiforme
hpi: hours post infection
IFN-γ: interferon-gamma
IL-4: interleukin-4
Infmed: infection medium
iNOS: nitric oxide synthase
LPS: lipopolysaccharide
MOI: multiplicities of infection
NO: nitric oxide
O/N: over night
OSC: organotypic slice culture
pfu: plaque forming unit
PI: propidium iodide
RFP: red fluorescent protein
rm: recombinant murine
VACV: vaccinia virus
w: with
w/o: without
